# *Ex vivo* investigation of a smart endotracheal tube for identifying esophageal intubation

**DOI:** 10.1117/1.JBO.30.6.067003

**Published:** 2025-06-13

**Authors:** Brett Gadsby, Sergiy Korposh, Ricardo Correia, Chenyang He, Barrie R. Hayes-Gill, Andrew M. Norris, Jonathan G. Hardman, David W. Hewson, Stephen P. Morgan

**Affiliations:** a University of Nottingham, Faculty of Engineering, Optics and Photonics Research Group, United Kingdom; b Medical Photonics Ltd, Nottingham, United Kingdom; c King Abdullah bin Abdulaziz University Hospital, Riyadh, Saudi Arabia; d Nottingham University Hospitals NHS Trust, Department of Anaesthesia and Critical Care, Nottingham, United Kingdom; e University of Nottingham, Division of Clinical Neurosciences, Department of Anaesthesia, United Kingdom; f Nottingham University Hospitals NHS Trust, School of Medicine, Nottingham, United Kingdom

**Keywords:** biosensor, endotracheal tube, optical fiber sensor, spectral reflectance, unrecognized esophageal intubation

## Abstract

**Significance:**

Unrecognized intubation of the esophagus instead of the trachea results in rapid and severe consequences for the patient. Utilizing the spectral properties of the tissues could reduce incidents of these events.

**Aim:**

We aim to investigate the design and implementation of a smart endotracheal tube (ETT) with integrated optical fiber sensors to distinguish esophageal and tracheal tissues.

**Approach:**

Computational methods are investigated to characterize and classify nine pairs of *ex vivo* porcine organs using spectral properties. Two classifiers [K-nearest neighbor and linear discriminant analysis (LDA)] are investigated.

**Results:**

Of the tissues sampled, 100% are correctly distinguished, with LDA being the preferred choice when considering both performance and applicability.

**Conclusions:**

In clinical practice, this approach offers a method for confirming correct tracheal intubation using the spectral properties of the tissues, performed in a single step with no other invasive medical device than the ETT required to detect the spectral measurements.

## 1 Introduction

Tracheal intubation secures a patient’s airway, delivering mechanical ventilation, oxygen, anesthesia, and medications. Unrecognized esophageal intubation (UOI) refers to the incorrect and unnoticed placement of the endotracheal tube (ETT) in the esophagus. Medical complications occur within minutes of UOI and can result in cardiac arrest, severe brain damage, or death due to the rapid onset of hypoxia.^1^^,^^2^ The current gold standard for confirming tube placement in the trachea is based on visual confirmation of the tube passing the vocal cords and the presence of a continuous carbon dioxide waveform from exhaled gas. However, visual confirmation is not always reliable, and ${\mathrm{CO}}_{2}$ waveform analysis can be compromised by low cardiac output, equipment issues, and misinterpretation, leading to incorrect tube placement identification in 5% to 15% of cases.^3^^,^^4^ These issues are particularly relevant in emergency treatment situations.^5^ Conditions such as obesity, swollen airways, limited neck mobility, and bleeding can further complicate airway management and increase the risk of UOI.^6^

Difficult airways are present in 4.6% to 23% of intensive care unit (ICU) patients,^7^^,^^8^ with this rising to 50% in the prehospital condition.^9^ The American Society of Anesthesiologists closed claims database showed that 3% to 8% of claims before 1990 were due to esophageal intubation, decreasing to 1% to 2% between 1990 and 2013.^10^^,^^11^ UK data from 1995 to 2007 estimated that 6% of anesthesia-related claims were due to UOIs.^12^ In 2016, UOI resulted in the deaths of two routine surgical patients in the UK,^13^^,^^14^ leading to its temporary addition to the NHS Never Events list. Fatalities from UOI have continued to occur as recently as 2020.^15^ This highlights the substantial risk of airway mismanagement, reinforcing the need for a more reliable intubation verification method.

A device that can integrate into a standard ETT and provide clear spectral evidence of UOI may greatly reduce patient harm. Previous research by Nawn^16^^,^^17^ using hyperspectral imaging and a bifurcated fiber optic probe in porcine and human cadaver models identified a spectral signature in the trachea in the 530 to 590 nm range, which is useful for detecting UOI. However, the routine use of a fiber optic scope is impractical. Integrating sensing technology within the ETT would allow immediate and automatic detection of incorrect placement during intubation. This study presents an innovative “smart” ETT that integrates fiber optic sensors, enabling real-time, automatic detection of UOI. Unlike previous approaches that rely on external ultrasonography,^18^ separate spectral probes,^16^ or additional clinical interventions,^19^ our design: (1) embeds spectral reflectance sensing directly into a standard ETT, eliminating the need for additional devices; (2) provides immediate classification of tracheal versus esophageal placement without requiring manual interpretation; and (3) leverages an optimized fiber sensor design that enhances light delivery and collection for robust spectral differentiation. This builds on our previous work developing a smart ETT for monitoring cuff pressure and tracheal perfusion *in vivo*,^20^ enhancing intra-tracheal multiplexed sensing by investigating whether the same sensor geometry used for photoplethysmography (PPG) measurements can be used to identify UOI.

To investigate this approach, we developed and tested a classification algorithm using *ex vivo* porcine trachea and esophagus samples. We investigated two machine learning classifiers: K-nearest neighbor (K-NN) and linear discriminant analysis (LDA) to distinguish among these tissue types. By integrating this automated detection system within the ETT, our approach eliminates the need for additional verification steps, improving patient safety in emergency, ICU, and prehospital airway management scenarios.

## 2 Methods

**Fig. 1:**
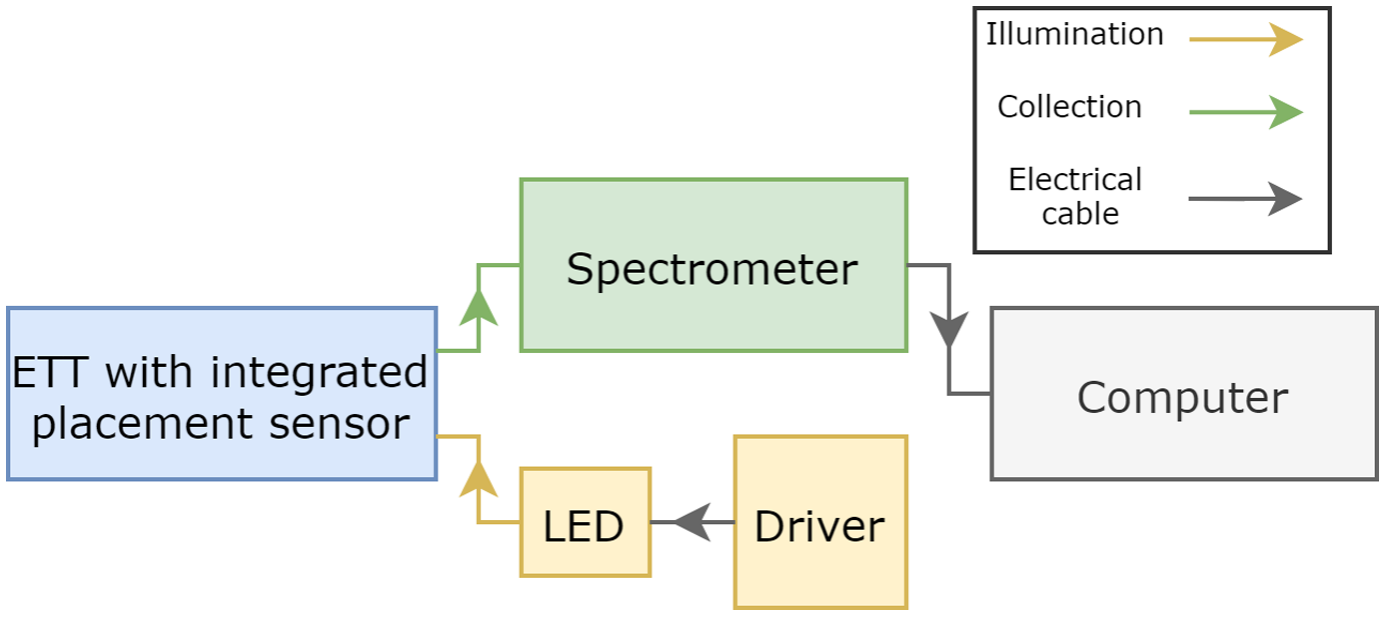
Opto-electronic instrumentation, with the fibers and units that provide illumination and collection of light shown in yellow and green, respectively.

This work was supported by the Medical Research Council, UK, under grant MR/T025638/1. The *ex vivo* animal samples were obtained from a local abattoir and involved no work with living animals. The investigation began with the fabrication of a two-optical fiber tube placement sensor and its integration into the cuff of an ETT. Figure 1 shows a schematic of the overall system in which white light from an LED is delivered into the smart ETT and the spectrum of the reflected light is analyzed. The smart ETT was placed inside nine tracheal and esophageal samples, and 50 spectra were acquired in each of the 18 organs. The two classifiers, K-NN^21^ and LDA,^22^ were then used to investigate whether tracheal and esophageal tissues can be distinguished.

### 2.1 Sensor Fabrication

**Fig. 2:**
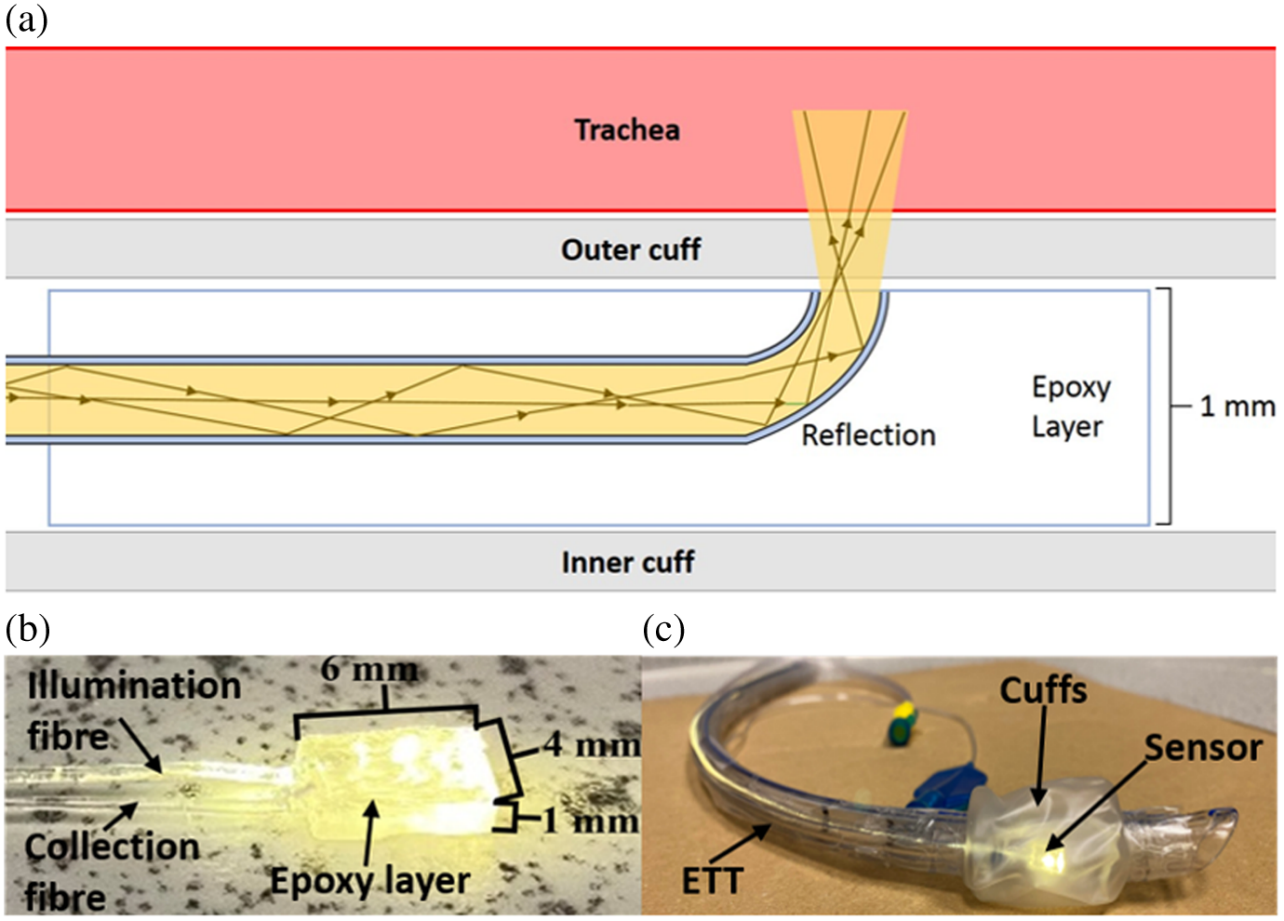
Operating principle and design of the tube placement sensor. (a) Schematic illustration showing the illumination fiber in an epoxy layer and how the light travels through the outer cuff and into the tissue. (b) Tube placement sensor containing J-shaped fibers. C) ETT with tube placement sensor between the cuffs.

Two plastic optical fibers [poly methacrylate (PMMA), Asahi DB-500, Tempe, Arizona, US] were bent to 90 deg under moderate heating to produce two J-shaped fibers. The fibers were bent into a J-shape so that the tips were flush and normal to the surface of the ETT cuff to maximize illumination and collection. The fibers were then placed into a 3D printed mold (dimensions 6×4×1  mm). A source–detector distance (SDD) of 3 mm was found to work well for the tracheal and esophageal samples, and this is consistent with our previous sensor SDD^20^ used for PPG measurements, where part of the study is to investigate whether the same sensor geometry can be used for tissue identification. Increasing the SDD also increases the penetration depth but reduces total returned optical power. The fibers’ bend was centralized, and then, two drops of UV-curable, transparent, biocompatible optical adhesive (Panacol, Vitralit, 1655, Torrington, Connecticut, US) were pipetted into the mold. The epoxy was then cured by a UV torch (UV395, Tattu, U1S, London, UK), emitting a power of 5 W with a central wavelength of 395 nm for ∼40  min. Although the curing time may affect the mechanical properties of the epoxy, it is not critical because it is the optical properties that impact the spectra. Afterward, the top of the sensor was then filed and polished to make it flush with the mold surface, resulting in sensor dimensions of 6×4×1  mm [Figs. 2(a) and 2(b)]. A size 8 Mallinckrodt cuffed ETT (107-80, Covidien, Watford, UK) with a high volume, low-pressure cuff made from polyvinylchloride was used in a “double cuff” arrangement (inner and outer cuff) described previously.^20^ The sensor was placed between the two cuffs and secured to the inside of the outer cuff with a drop of the optical adhesive Fig. 2(c).

### 2.2 Opto-Electronic Unit

The illumination fiber of the sensor was coupled to a broadband LED with a wavelength range of 470 to 850 nm (Fiber-Coupled LED, Thorlabs, MBB1F1, Newton, New Jersey, United States). The LED driver was set to deliver a drive current of 500 mA with a current ripple of 8 mA (T-Cube LED Driver, Thorlabs, LEDD1B, Newton, New Jersey, US). The detection fiber was coupled to a UV–visible spectrometer (USB2000+UV−VIS−ES, Ocean Optics, Orlando, Florida, United States), set to a 200 ms integration time, a BoxCar width of 1 (no wavelength averaging), and with no scan averaging. The spectrometer was connected to a Windows PC via USB for data acquisition and to supply power. All fiber connectors were universal bare fiber terminators (Thorlabs, BFT1, Newton, New Jersey, US), with 0.5 mm SubMiniature A (SMA) multimode connectors (B10125A—SMA905, Thorlabs, Newton, New Jersey, US).

### 2.3 Spectra Acquisition

**Fig. 3:**
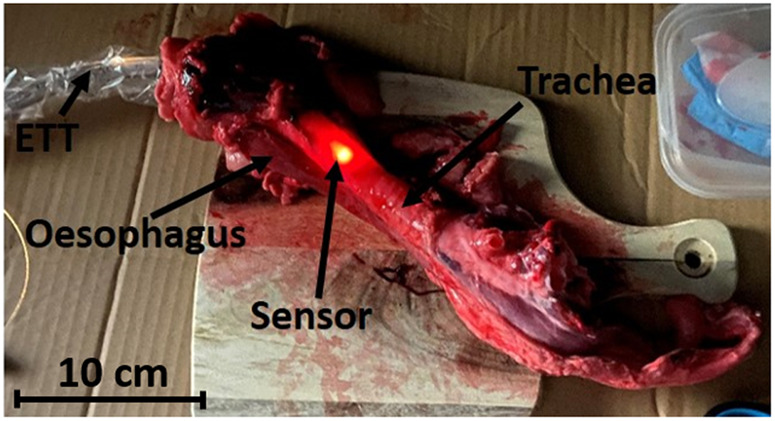
Porcine trachea and esophagus sample with smart ETT placed through the larynx and into the trachea.

Nine porcine (Tamworth breed, female) trachea and esophagi were obtained from a local abattoir, and each sample consisted of the connected trachea and esophagus, from the larynx to the vessels surrounding the heart (Fig. 3). The ETT was placed in a transparent medical sensor sleeve (Pegasus Surgi Safe Tubing Sleeve, small (18×1.75), Dental Sky, SKU: 50-137, UK) so that the fluids from the sample did not have to be cleaned from the ETT with each intubation. The ETT with integrated placement sensor was first covered with a nontransparent black felt cover, and a dark reference spectrum was taken. A white reference spectrum was then taken by placing the sensor inside the ETT cuff and medical sensor sleeve, against a white reference standard (Spectralon diffuse reflectance target, Labsphere, AA-00827-000, Sutton, Massachusetts, US). The light and dark references were then used to normalize the signal and remove background noise. For each sample, the smart ETT was first passed through the larynx and vocal cords and into the trachea. The first spectrum was taken when the ETT cuff had just passed the vocal cords. Each sequential spectrum was then taken after further inserting and rotating the ETT. The distance of each further insertion was ∼4  mm, ensuring that the sensor was positioned alternatively on the hyaline cartilage rings and between them so that an even distribution of tissues was acquired. Each ETT rotation was ∼20  deg, ensuring data acquisition from the posterior, left and right lateral, and anterior. When the tip of the ETT reached the bottom of the trachea (Carina), it was retracted and rotated out of the trachea similarly to insertion until 50 spectra were obtained. The sensor sleeve was then replaced, and the ETT was placed into the esophagus, where the same method of insertion and rotation was used until 50 spectra were again acquired. The ETT was then removed, the sensor sleeve replaced, and the next samples measured until all nine trachea and esophagi were completed and a total of 900 spectra were acquired.

## 3 Results

### 3.1 Spectral Processing

**Fig. 4:**
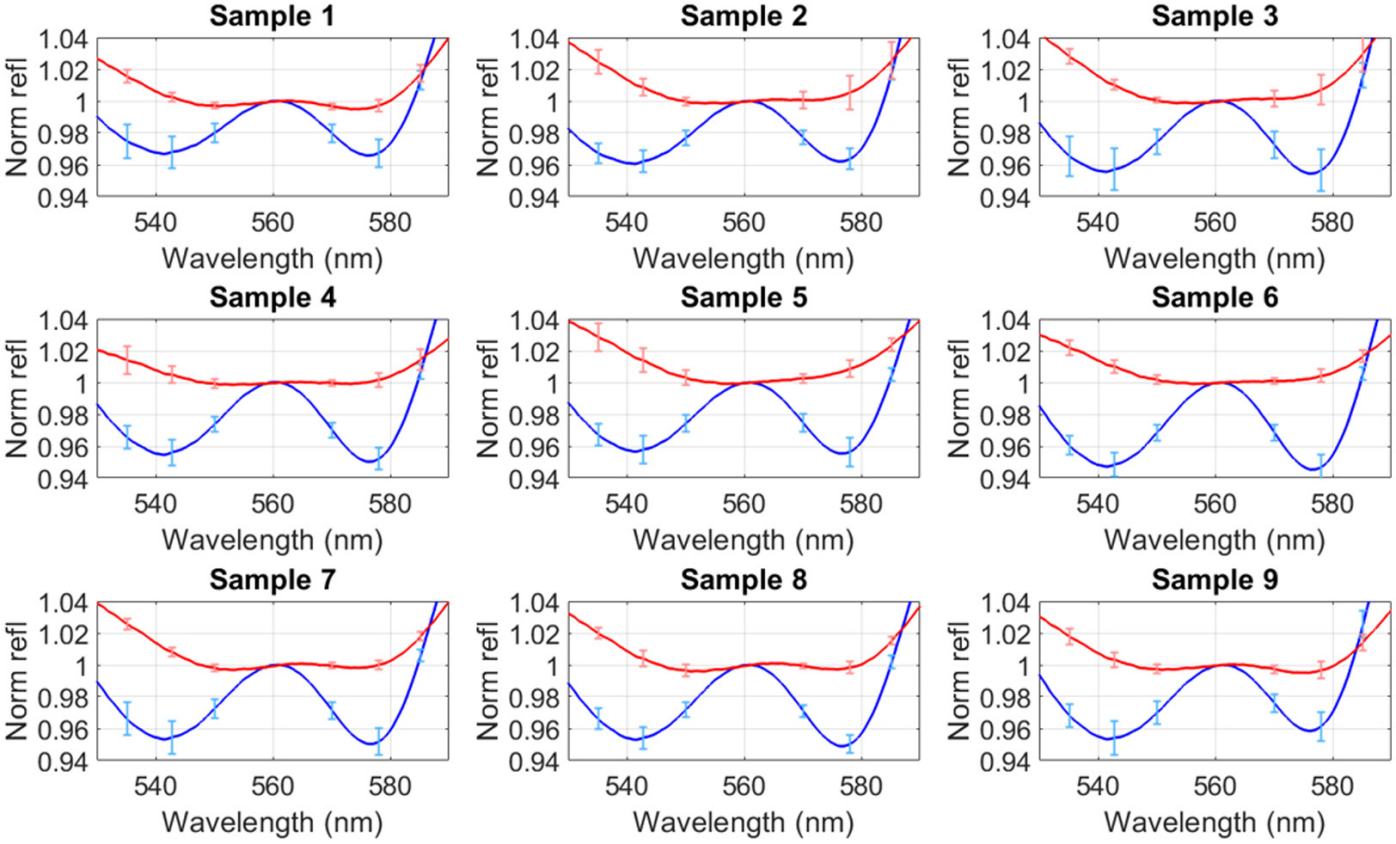
Mean tracheal (blue) and esophageal (red) normalized reflectance (norm refl) of the 50 spectra for samples 1:9. The spectra have been normalized such that the value at the central peak of 561 nm is 1. The error bars are found from the standard deviations of the 50 normalized trachea and esophagi spectra at wavelengths 535, 543, 550, 570, 578, and 585 nm and are shown in the blue and red error bars, respectively.

The tracheal and esophageal spectra of the nine samples are shown in Fig. 4. The spectra have been normalized at the central peak (561 nm), resulting in the variance mostly being expressed in the two reflectance troughs (543 and 578 nm), providing two variables to distinguish the two tissues. The tracheal characteristic consisting of a trough-peak-trough at 543, 561, and 578 nm can be seen in the trachea spectra (blue spectra in Fig. 4). Although this characteristic is present in the esophagus sample, it is less pronounced (red spectra in Fig. 4).

### 3.2 *K*-Nearest Neighbor

By combining the reflectance values at both characteristic wavelengths (543 and 578 nm) and plotting them against each other, a classification using both wavelengths can be performed.

**Fig. 5:**
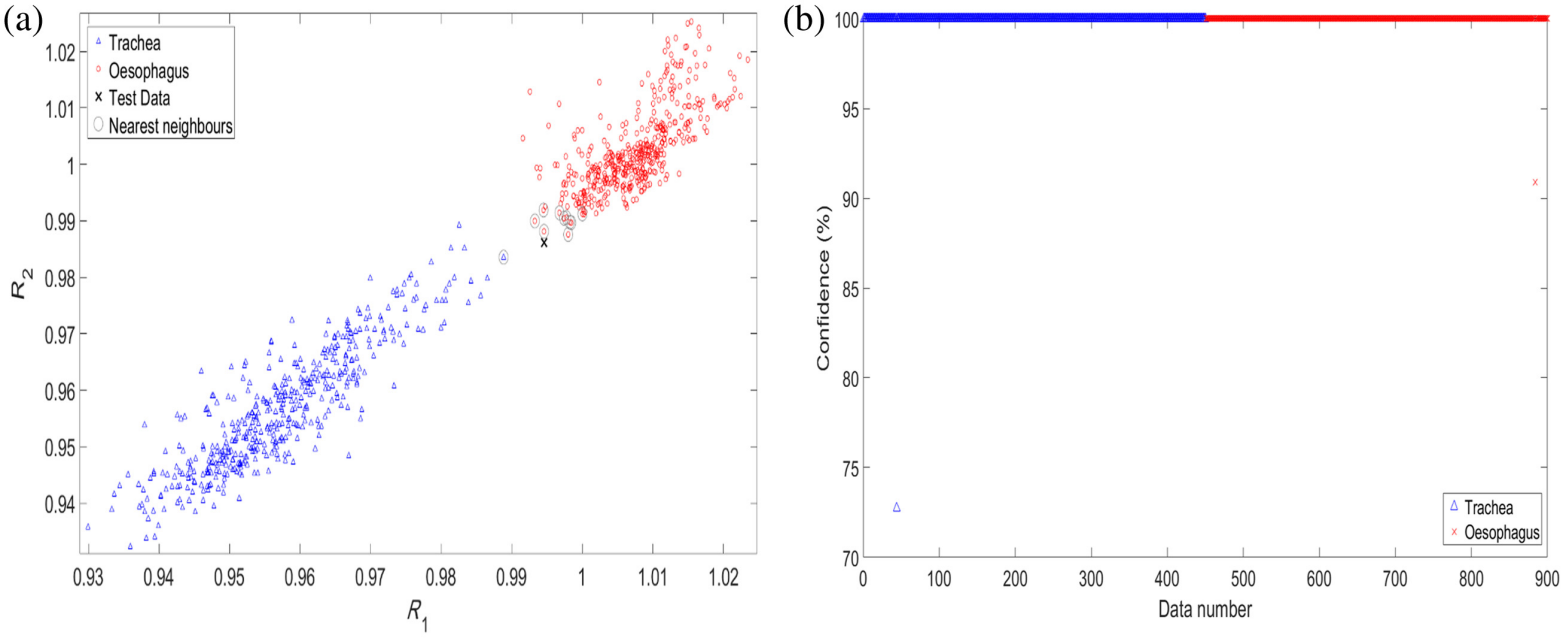
(a) Example of the Minkowski K-NN classifier on a single data point, with the 10 nearest neighbors highlighted with a circle around the data point. (b) Percentage confidence of the K-NN classifier for all trachea (blue) and esophagus (red) data.

For the test data point labeled “X” in Fig. 5(a), there are eight tracheal neighbors (including itself) and two esophageal neighbors, so the test data would be classified as trachea, with 8/11 (72.7%) confidence. From Fig. 5(b), the K-NN classifier correctly identifies 100% of the data, with seven data points not having their 10 nearest neighbors as the correct tissue and therefore not being classified with 100% confidence. The lower limit of the 95% confidence interval for the probability of correct identification when all placements are identified correctly in the 900 observations is 99.6%. Data in Fig. 5(b) are plotted as discrete points but appear as a straight line due to the high proportion of points being identified with 100% confidence. The mean-square error (MSE) on the K-NN classifier for the full cohort of data was found to be $7.9\times 10^{-7}$. The peak signal-to-noise ratio (PSNR) was found to be 69.5 dB.

### 3.3 Linear Discriminant Analysis

**Fig. 6:**
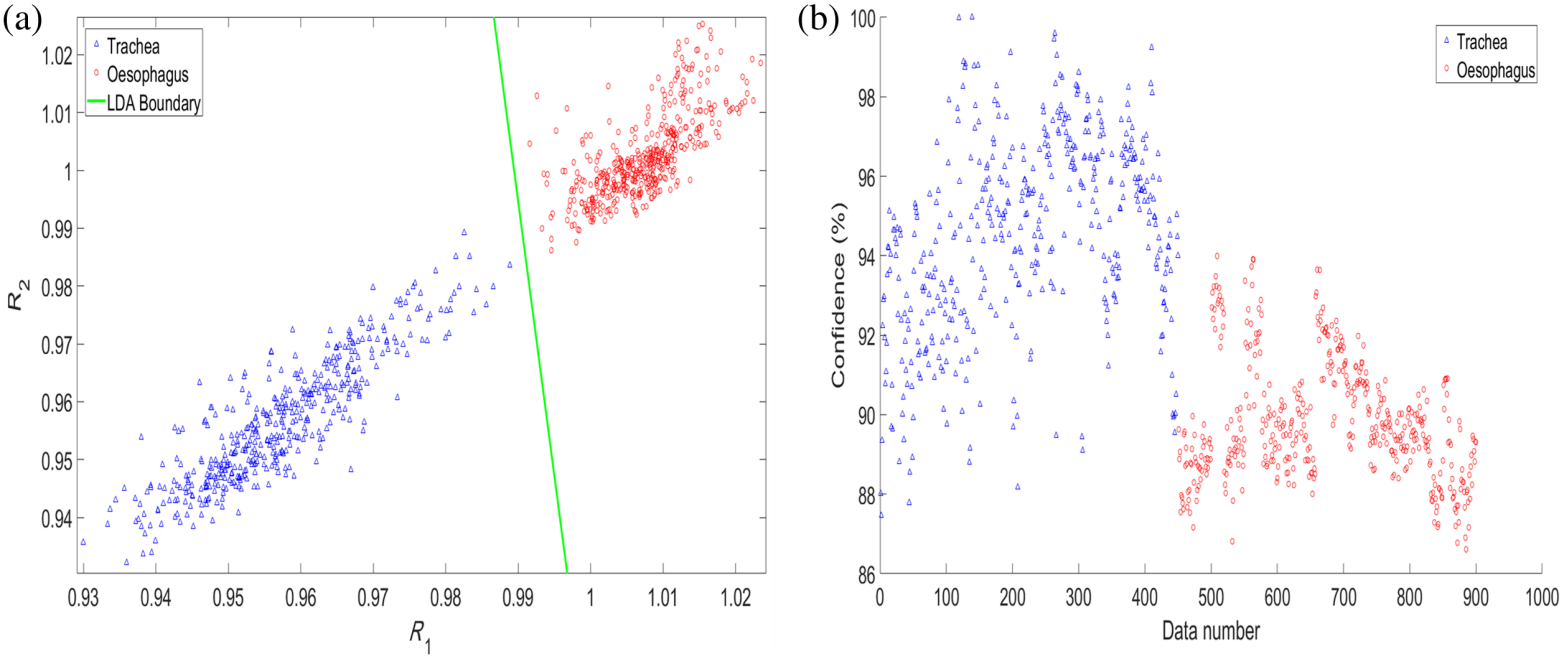
(a) Scatter plot of the trachea (blue triangle) and the esophagus (red circles) normalized reflectance data, with an LDA boundary (green line). (b) Percentage confidence of the LDA classifier for all trachea (blue) and esophagus (red) data.

By performing LDA on the data, a boundary between the two tissue types can be found [Fig. 6(a)], and 100% of the tissues can be correctly classified. If a data point is to the left (below) of the line, it is classified as tracheal, if the data point is to the right (above) of the line it is classified as esophageal. The lower limit of the 95% confidence interval for the LDA approach is 99.6%. Figure 6(b) shows the confidence on each data point, where it has been scaled such that the data point furthest from the line gives a 100% confidence. The MSE on the K-NN classifier for the full cohort of data was found to be $7.4\times 10^{-3}$. The PSNR was found to be 21.3 dB.

## 4 Discussion

Using appropriate classifiers, 100% of the tissue data samples can be correctly classified by both the K-NN and LDA methods. Although it was found that the first normalized wavelength (543 nm) could correctly identify 100% of the tissues, combining this with the second wavelength (578 nm) and prospective classifier will likely yield a better classification in less separable data, such as that from *in vivo* research. For LDA, the weighting function amplified values close to the boundary, but realistic confidences require more *in vivo* research to determine the optimum weighting. Figure 6(b) was produced by calculating the distance from each data point to the LDA boundary and then scaling the distance exponentially such that data closer to the line have higher percentage confidence, with the furthest data point from the line giving 100% confidence and the data closest to the boundary giving a 63% confidence.

For both the K-NN and LDA classifiers, the lower limit of the 95% confidence interval for the probability of correct identification is 99.6% as all placements are identified correctly in the 900 observations. This calculation assumes that all observations are independent, although we recognize that this is not the case. Due to this, the lower limit of the confidence interval calculated in this paper is higher than in reality. However, as this is a proof-of-concept study, these results provide good evidence that this method should proceed to *in vivo* research or clinical trials where natural misclassifications are more likely to occur, which will yield a stronger statistical basis for comparison.

The K-NN model achieved an MSE of $7.9\times 10^{-7}$ and a PSNR of 69.5 dB, indicating extremely high accuracy and confidence, which is to be expected given the high separability of the data with high confidence. The LDA classifier produced an MSE of $7.4\times 10^{-3}$ and a PSNR of 21.3 dB. Furthermore, it reflects a global decision boundary that generalizes across the dataset with lower confidence. LDA also maintains a linear boundary throughout the data set, making it more sensitive to class overlap and feature variability. Although K-NN benefits from local adaptability, increasing the number of neighbors would reduce classification confidence, especially on those closer to the boundary. This may yield a more representative confidence, leading to a higher MSE and lower PSNR.

Although the selected 3 mm SDD was chosen due to its similarity to pulse oximetry techniques and through previous experimentation when using the probe for PPG measurements^20^ for applications in the trachea, the optimal SDD can vary significantly depending on tissue properties and measurement conditions. Previous studies have shown that diffuse reflectance spectroscopy is highly susceptible to movement artifacts and pressure variations, requiring careful geometry optimization to achieve robust *in vivo* measurements.^23^ If an *in vivo* investigation results in unsatisfactory findings, then future improvements could involve more systematic testing of different SDD configurations to assess penetration depth and sensitivity to variations in tissue optical properties. Although the current normalization process partially mitigates variability in absolute absorption using a reflectance ratio, alternative normalization techniques, such as multiwavelength calibration or machine learning-based correction, could further enhance consistency.^24^ In addition, the observed variation in spectral intensity across the 50 acquired spectra is attributed to differences in probe-tissue contact and subtle anatomical heterogeneity at different tissue positions. Despite these variations, normalization produces consistent spectral shapes (Fig. 4), and the chosen ratio wavelengths remain highly separable (Figs. 5 and 6).

Both LDA and K-NN perform well, but LDA can be more easily and rapidly applied. During clinical use of the smart ETT, only a single straight-line equation would be stored and compared with an unknown tissue measurement, as opposed to the K-NN method where every training data point needs to be compared with the unknown. Although the K-NN confidences presented [Fig. 5(b)] are higher than the LDA’s [Fig. 6(b)], these were chosen only as examples, and the scaling of them would be modified once more data are acquired. Other classifiers such as logistic regression analysis^25^ and support vector machines^26^ were also investigated (data not shown here) and proved successful in distinguishing the two tissue types. However, they are both more computationally expensive to implement than K-NN and LDA and yield similar success rates. These may be explored further in future research where the data are less easily classified, such as with *in vivo* measurements in the presence of blood and aspiratory fluids from the trachea and esophagus.

The presence of the tracheal characteristic has been documented previously by Nawn.^16^ This research provides a more practical and convenient application of this characteristic in the form of smart ETT that can identify incorrect intubation in a single step without further clinical intervention such as the use of an additional fiber optic probe. The smart ETT incorporates a small optical reflectance sensor into a standard ETT, along with the prospective classifiers to distinguish the tissues. Further advantages include utilizing an LED, which produces a high proportion of light in the relevant wavelength region of 530 to 590 nm, a J-shaped sensor design to launch and collect light more efficiently to/from the tissue, and integration of the sensor into the cuff. The latter brings the sensor into intimate contact with the tissues, thus reducing motion artifacts and achieving high-quality signals,^27^ supporting better classification. Principal component analysis (PCA) has been demonstrated by Nawn^17^ to help distinguish the two tissue types. However, due to the normalization of the spectra at the central peak (561 nm), the variance is mostly expressed in the two troughs of the characteristic (578 and 543 nm), and dimensionality reduction is unnecessary. The similarity between the two spectra types and the first two principal components is less desirable to distinguish the tissues when compared with the classifiers explored in this paper due to their complexity and success. PCA may be of use in future research when other variables are measured alongside the spectral characteristics, such as blood perfusion, oxygen saturation, and interface pressure between the cuff and the tissue.^16^ These variables may be used to help distinguish the two tissues, where PCA can reduce the dimensions of the matrix into a smaller number of uncorrelated variables, which maximizes variance and increases interpretability, while still retaining most of the information.

The origin of the tracheal reflectance characteristic has been suggested to originate from the presence of oxyhemoglobin.^16^ Due to its similarity to the extremely well-documented absorption characteristic, the characteristic does clearly originate from oxyhemoglobin.^28^^,^^29^ The thinner more transparent composition of the tracheal mucosa along with the white reflective cartilage likely causes incident light to more readily interact with the oxygenated blood, whereas the thicker, less transparent mucosa of the esophagus scatters and absorbs the light. Therefore, tears in the esophageal lining could allow the light to penetrate the mucosa and the muscular layer and potentially result in false negative results.

Blood contamination during intraoperative intubation could present a significant challenge for diffuse reflectance spectroscopy due to the strong absorption features of oxygenated hemoglobin at 543 and 578 nm, the same wavelengths used to identify tracheal characteristics. Previous work by Berard^30^ demonstrated that the presence of blood can alter spectral reflectance, potentially masking tissue-specific features. However, despite this interference, it was shown that tracheal spectral characteristics remained distinguishable, albeit with reduced classification confidence. One approach to mitigating the effects of blood is to incorporate multiwavelength spectral correction techniques, such as differential normalization using reference wavelengths that are minimally affected by hemoglobin absorption. In addition, temporal averaging of spectra during continuous measurements may help minimize transient variations due to blood pooling. Further improvements could involve employing machine learning models trained on datasets with controlled blood contamination to identify and compensate for hemoglobin-induced distortions in real time. Moreover, alternative illumination strategies, such as angled light delivery or polarization filtering, may help reduce specular reflections from pooled blood and enhance tissue-specific signals. Although the current study did not explicitly investigate the effects of blood interference, future *in vivo* work should incorporate controlled bleeding conditions to optimize the robustness of this approach in clinical environments. Multiple sensors and a voting system could also reduce false negatives.

Future work will focus on the repeatability of multiple sensors, to determine if a calibration of each sensor is necessary to distinguish the two tissues accurately, or if a single boundary can be found that satisfies new sensors. *In vivo* experimentation will also reveal if the sensor can perform under more clinically applicable conditions.

## 5 Conclusion

UOI can result in cardiac arrest, severe brain damage, or death due to hypoxia by asphyxiation. A new smart ETT has been demonstrated with integrated optical fiber sensing to indicate UOI in a single step without additional clinical intervention. By utilizing the spectral properties of the trachea, acquired with a two-fiber sensor integrated into the cuff of a standard ETT, *ex vivo* tracheal and esophageal porcine tissues can be well distinguished with 100% success for nine pairs of samples and 50 measurements per sample. The *K*-NN and LDA classifiers have been highlighted due to their ease of implementation and rapid processing, although other classifiers perform well due to the high separability of the data.

## Data Availability

The code and data presented in this article are publicly available in figshare at: https://figshare.com/s/793e26db41b547b37d08
